# Applications of Biological Therapy for Latent Infections: Benefits and Risks

**DOI:** 10.3390/ijms25179184

**Published:** 2024-08-24

**Authors:** Yuan Zong, Koju Kamoi, Miki Miyagaki, Jing Zhang, Mingming Yang, Yaru Zou, Kyoko Ohno-Matsui

**Affiliations:** Department of Ophthalmology & Visual Science, Graduate School of Medical and Dental Sciences, Tokyo Medical and Dental University, Tokyo 113-8510, Japan; zongyuan666@gmail.com (Y.Z.); miyagakimk@gmail.com (M.M.); zhangj.c@foxmail.com (J.Z.); yangmm-12@outlook.com (M.Y.); alicezouyaru519@gmail.com (Y.Z.); k.ohno.oph@tmd.ac.jp (K.O.-M.)

**Keywords:** biological therapy, latent infection, pathogen reactivation, risk management

## Abstract

Biological therapies have revolutionized medical treatment by targeting the key mediators or receptors involved in inflammatory responses, thereby effectively suppressing inflammation and achieving beneficial outcomes. They are more advanced than conventional therapies using corticosteroids and immunosuppressants, offering effective solutions for autoimmune diseases, cancer, transplant rejection, and various infectious diseases, including coronavirus disease 2019. Although they exert low immunosuppressive effects, biological therapies can reactivate specific biological targets associated with infections. This review summarizes the currently available biological therapies and discusses their immunosuppressive mechanisms and clinical applications, highlighting the variations in the types and frequencies of infection recurrence induced by different biological agents. Additionally, this review describes the risk factors associated with various biological agents, thus aiding clinicians in selecting the most appropriate biological therapy.

## 1. Introduction

Biological therapies primarily target key mediators or receptors involved in inflammatory responses and effectively inhibit the occurrence and progression of inflammation to achieve therapeutic goals. These therapies have revolutionized the field of medicine, offering effective treatments for autoimmune diseases, tumors, transplant rejection, and various infectious diseases, including coronavirus disease 2019 (COVID-19). They are more advanced than conventional therapies involving corticosteroids and immunosuppressants [1,2,3,4]. The currently available biological therapies, their immunosuppressive mechanisms, and clinical applications are summarized in Table 1. Despite exhibiting lower immunosuppressive effects than those of the other treatment modalities, biological therapies may induce specific biological targets related to infection [5]. For example, tumor necrosis factor (TNF)-α inhibitors may lead to tuberculosis reactivation, and anti-CD20 targeted therapy may lead to hepatitis B virus (HBV) reactivation [6,7,8,9]. Different biological agents exhibit clinical variations in the types and frequencies of latent infectious pathogen reactivation. This review discusses the risk factors and diseases associated with various biological agents to help clinicians in selecting the most appropriate biological therapy for patients. Specifically, this article focuses on the risks of latent infectious pathogen reactivation associated with different biological agents and suggests preventive measures against various infections, such as tuberculosis (TB) and chronic viral infection reactivation.

## 2. Mycobacterium Tuberculosis (MTB)

### 2.1. Pathogenesis and Latency

One third of the global population harbors latent TB infection (LTBI) caused by MTB. In individuals with a competent immune system, MTB is engulfed by the alveolar macrophages, leading to the formation of granulomas via the activation of macrophages and T cells. This containment strategy keeps bacteria dormant without overt clinical manifestations, leading to LTBI [10,11]. However, in cases of immunosuppression, the ability of the immune system to maintain the integrity of granulomas diminishes, allowing latent MTB to escape and proliferate, leading to active TB infection [12,13]. 

### 2.2. Biologics and Reactivation

TNF-α inhibitors can reactivate LTBI by inhibiting the activity of TNF-α, a critical cytokine involved in the formation and maintenance of granulomas encapsulating the bacteria and activation of macrophages responsible for the phagocytosing and killing of MTB, thereby weakening the ability of the immune system to control latent bacteria, causing the breakdown of granulomas, reducing the efficacy of macrophages, and ultimately allowing the dormant bacteria to escape and proliferate, resulting in active TB infection [14,15]. TNF inhibitors significantly increase the risk of TB [14,15,16,17,18,19,20]. Therefore, comprehensive screening, including tuberculin skin tests, is necessary to rule out MTB infections in patients undergoing treatment with biological agents.

Antigen-presenting cells (APCs) induce Th17 cell differentiation and promote the production of interleukin (IL)-17 and IL-22. IL-23 and IL-17 serve as critical targets for autoimmune diseases, such as psoriasis, and form an integral part of immune prophylaxis against TB [21]. To date, no clinical trials and real-world studies have reported TB reactivation in patients with treated or untreated LTBI exposed to IL-23 inhibitors [11,22,23,24,25]. Blauvelt et al. documented a case of bone TB in a 58-year-old Asian male patient following the administration of tildrakizumab (200 mg), ultimately necessitating treatment discontinuation; the patient initially tested negative for TB in the single-step Mantoux test [23]. Sporadic cases of active TB have been reported following the introduction of the IL-12/IL-23 dual inhibitor, ustekinumab, in clinical therapy, regardless of the administration of other anti-tuberculosis prophylaxes [10,26,27,28]. In a Phase III trial of ustekinumab, involving five clinical studies conducted in North America, Europe, and Asia, 3177 patients with psoriasis were evaluated. Among these patients, 167 with LTBI did not develop active TB after receiving isoniazid prophylaxis [29].

Anti-IL-17 therapies, including secukinumab and ixekizumab, exhibit a low risk of MTB reactivation. In a retrospective, multicenter, and multinational study, the tolerability and safety of IL-17 and IL-23 inhibitors were evaluated in patients with psoriasis with newly diagnosed LTBI who received no treatment and in those who received chemoprophylaxis for TB. In this study, only one patient who had not undergone TB screening and did not receive TB chemoprophylaxis was diagnosed with intestinal TB after 14 months of treatment with the IL-17 inhibitor, ixekizumab [11]. To date, no cases of MTB reactivation have been reported in other clinical trials and real-world studies [11,23,30].

Similar to TNF-, IL-6 is a multifunctional pro-inflammatory cytokine involved in a wide range of inflammatory responses and immune regulation, including immune reactions, synovial inflammation, hematopoiesis, and the transition from acute to chronic inflammation [31]. Anti-IL-6 receptor therapy can alleviate various symptoms related to the immune and inflammatory systems owing to its diverse roles in immune and inflammatory system dysfunction. Following a post-marketing research evaluation of all rheumatoid arthritis (RA) cases, the incidence rate of TB after exposure to the IL-6 receptor antibody tocilizumab (TCZ) was found to be 0.05% [32]. Clinical reports have been documented predominantly in countries with a high risk of TB. In a post-marketing surveillance study conducted in Japan in 2008, 3881 RA patients receiving TCZ treatment at a dose of 8 mg/kg every 4 weeks were monitored for 28 weeks, during which four new cases of active TB were observed [33]. Schiff et al. analyzed the cumulative safety data from eight clinical trials of TCZ; among the 4009 patients who received at least one dose of TCZ, seven trials reported eight cases of MTB infection, all of which were newly diagnosed and originated from countries with a high risk of TB [34]. In a retrospective investigation by Itagaki et al., patients treated with biologics and with a history of anti-mycobacterial therapy at Keio University Hospital in Japan from 1 January 2012 to 31 August 2020 were reviewed. This study included 44 individuals who had an established risk of developing TB before initiating TCZ treatment and commenced LTBI therapy as a preventive measure. Among them, two patients developed TB after starting LTBI treatment, both testing positive for interferon-gamma release assays (IGRAs) in TB screening tests [35]. 

During the COVID-19 pandemic, TCZ was widely used to manage patients with severe COVID-19 infections exhibiting elevated systemic inflammatory marker levels. NOH et al. documented a case of active TB emerging after TCZ administration for COVID-19 [36]. Overall, the risk of TB reactivation with TCZ is relatively low; however, proper screening before use, the implementation of preventive therapy measures, and close monitoring during treatment are essential for risk management. 

Owing to its immunosuppressive effects, similar to those of anti-TNF therapy, the Janus kinase (JAK) inhibitor tofacitinib (CP-690,550) also increases the risk of infection, including with TB [37]. Experimental evidence indicates that tofacitinib diminishes the ability of the host to control TB in animal models while enhancing the reactivation of LTBI [38]. Winthrop et al. reviewed the phase II, phase III, and long-term extension clinical trial data of tofacitinib for RA before April 2013. They found that among 5671 patients treated with tofacitinib, 26 cases of active TB were reported, making it the most common opportunistic infection; most TB cases occurred in patients receiving higher doses of the medication. Of the 26 cases, 21 (81%) occurred in countries with a high TB burden [37]. Before initiating tofacitinib therapy, it is essential to conduct screening and treatment for LTBI, particularly in regions with a high prevalence of TB, similar to the approach used with anti-TNF therapies.

Abatacept (CTLA4-Ig), classified as a T-cell stimulation blocker, binds to the co-stimulatory molecules CD80/86 on APCs and CD28 on T cells, thereby inhibiting T-cell activation [39]. Recent studies have revealed the crucial role of the CD28 signaling pathway in combating MTB infections [9,40]. However, current clinical reports indicate that active TB in patients treated with abatacept is a rare event, with a significantly lower incidence than that in patients with RA treated with TNF inhibitors [9,41].

In conclusion, the risk of LTBI reactivation with biological therapies is evident, particularly in patients residing in regions with high TB prevalence or in those with additional TB risk factors. The most significant and well-documented risk is associated with anti-TNF therapy, whereas the risk associated with other non-anti-TNF biologics is relatively low. Nevertheless, before initiating biological therapy, physicians should assess the TB risk of the patient, and, if necessary, conduct TB screening and prophylactic treatment to ensure patient safety and well-being.

## 3. HBV

### 3.1. Pathogenesis and Latency

HBV belongs to a family of hepatotropic DNA viruses and is highly prevalent in Asia and Africa. HBV can result in acute and chronic hepatitis, which may progress to liver cirrhosis or hepatocellular carcinoma [42]. Globally, approximately 300 million people are chronically infected with hepatitis B, with more than 500,000 deaths annually from HBV-related diseases [43]. In adults, >95% of HBV infections are self-limiting; however, replication-competent HBV DNA can persist in the liver for several years or even decades. HBV reactivation (HBVr) can occur in patients with evident chronic infection (hepatitis B surface antigen [HBsAg]-positive) and in patients with past markers of HBV infection, as evidenced by the presence of antibodies to the hepatitis B core antigen (anti-HBc) with or without anti-HBs, in the absence of circulating HBsAg [44]. Immunosuppressive therapies, including biologics, can induce immunosuppression, allowing the reactivation of a previously dormant or low-replicating HBV, leading to HBVr [42]. The HBV infection status is closely linked to the risk of HBVr. The risk of hepatitis B reactivation is significantly increased by 5–8 fold in patients who are positive for HBsAg compared with those who are negative for HBsAg but positive for anti-HBc [45,46].

### 3.2. Biologics and Reactivation

Table 2 summarizes the risk of HBV reactivation faced by the planned use of biological therapies based on the baseline HBV status. Anti-CD20 antibodies, such as rituximab, deplete B lymphocytes by binding to CD20 surface markers. This depletion or dysfunction of B cells may impair the activation and proliferation of CD4-positive T cells specific to HBV, ultimately leading to HBVr infection [6,47]. Evens et al. conducted a meta-analysis of rituximab-associated HBVr cases reported in the United States Food and Drug Administration (FDA) Adverse Event Reporting System (FAERS) MedWatch database from 1997 to 2009. Their findings indicated that patients receiving rituximab-containing therapies had an over 5-fold-increased incidence of HBVr compared with those receiving non-rituximab treatments [48]. In another meta-analysis, conducted by Paul et al., the risk of reactivation was higher in patients who received rituximab-containing chemotherapy. Specifically, among 800 patients receiving rituximab-containing therapies from 10 studies, the reactivation risk was 10% (95% confidence interval [CI] 5.8–16%), compared with a 4.0% risk (95% CI 2.2–6.3%) in 580 patients from eight studies involving non-rituximab chemotherapy [49]. Other anti-CD20 monoclonal antibodies, such as ofatumumab, exert their effects through similar mechanisms [47,50]. Therefore, all patients undergoing anti-CD20 immunochemotherapy should be screened for HBVr. Patients who test positive for HBsAg or HBcAb should begin prophylactic anti-HBV nucleoside therapy (NAT) to prevent HBVr [47,51]. Data from the Phase III GOYA and GALLIUM studies indicate that prophylactic NAT can effectively prevent HBVr in patients with a history of hepatitis B undergoing treatment with obinutuzumab and rituximab, reducing the risk by 91% [50].

Studies using in vitro and animal models have demonstrated that TNF can inhibit HBV replication and stimulate HBV-specific T-cell responses, suggesting a vital role for TNF in the clearance of HBV from infected hepatocytes [52,53]. Consequently, the use of anti-TNF monoclonal antibodies may enable HBV to evade host antiviral defense mechanisms. A systematic review and analysis of case reports indicated that HBVr occurred in approximately 40% (35/89) of HBsAg-positive patients, and approximately 5% (9/168) of HBsAg-negative and anti-HBc-positive patients receiving anti-TNF therapy [54]. Recent meta-analyses have shown that HBsAg-negative/anti-HBc-positive patients receiving anti-TNF therapy without NAT prophylaxis have an HBVr risk of 1% [10]. It is widely recognized that individuals who test positive for HBsAg should receive prophylactic measures. Conversely, patients who are negative for HBsAg but positive for anti-HBc may be evaluated on a case-by-case basis, contingent on the given circumstances [55]. 

For cytokine inhibitors targeting factors other than TNF, a meta-analysis indicated that the overall risk of HBVr for HBsAg-positive patients not receiving prophylaxis is high (35.5%), while the risk for HBsAg-negative, anti-HBc-positive patients is moderate (7%) [10,56].

Mogamulizumab (Moga) is a humanized monoclonal antibody targeting C-C chemokine receptor type 4 (CCR4). In the United States, the FDA approved mogamulizumab in 2018 for the treatment of relapsed or refractory mycosis fungoides and Sézary syndrome [57]. In the FAERS database, eight documented cases of HBVr are linked to mogamulizumab use compared to the 2290 cases linked to other drugs. The calculated reporting odds ratio (ROR) was 143.67, with *p* < 0.001 and 95% CI of 71.17–290.04 [57].

Current research on the impact of other biological therapies that directly suppress T cells/B cells on HBVr, particularly in HBsAg-positive patients, remains sparse. A retrospective cohort study observed that four out of eight (50%) HBsAg-positive RA patients experienced HBVr following JAK inhibitor baricitinib therapy. In the same study, out of 207 HBsAg-negative/HBcAb-positive patients, 30 (14%) had HBVr [58]. Another retrospective cohort study revealed that among 116 patients with RA treated with the JAK inhibitor tofacitinib, only two HBsAg-positive patients who did not receive prophylactic treatment experienced HBVr, representing 50% of this subgroup [59]. 

A recent systematic review analyzed eight studies encompassing 1057 HBsAg-positive patients receiving immune checkpoint inhibitors and reported that HBVr occurred in 18 (1.7%) patients. Notably, the reactivation rate was approximately 11% among 56 patients who did not receive prophylactic HBV treatment [60]. 

A meta-analysis of four studies estimated the risk of HBVr associated with abatacept as approximately 40% in HBsAg-positive patients and 5% in HBsAg-negative anti-HBc-positive patients who did not receive NAT prophylaxis [60].

In conclusion, HBVr is a significant risk factor for biological therapies, particularly anti-CD20 and anti-TNF treatments. Prophylactic anti-HBV NAT is crucial for HBsAg-positive patients. Emerging biologics, including JAK and immune checkpoint inhibitors, also pose a risk for HBVr. Therefore, comprehensive HBV screening and prophylaxis are essential to ensure patient safety during biological therapy.

## 4. Hepatitis C Virus 

### 4.1. Pathogenesis and Latency

Approximately 71 million people worldwide suffer from chronic Hepatitis C Virus (HCV) infection, with reactivation predominantly occurring in immunosuppressed individuals [61]. Typically, in patients with chronic HCV infection, the levels of HCV-RNA fluctuate within a stable range of approximately 0.5 log_10_ IU/mL, and HCVr is defined as an increase in HCV-RNA of ≥1 log_10_ IU/mL compared to baseline levels [61,62]. Approximately 23% of cancer treatment recipients experience HCV reactivation [62]. In comparison to HBVr, the clinical course associated with HCVr is generally milder [62]. 

### 4.2. Biologics and Reactivation

Patients with chronic HCV infection are at risk of viral reactivation or acute exacerbation following the initiation of anti-CD20 therapy [63]. Currently, there is insufficient evidence to suggest that anti-TNF treatment increases the risk of viral reactivation in patients with chronic hepatitis C. Nevertheless, caution should be exercised when administering these medications [63,64,65]. Patients should be closely monitored throughout the course of biological therapy.

## 5. Herpesviruses

### 5.1. Pathogenesis and Latency

Varicella zoster virus (VZV) belongs to the herpesvirus family and is prevalent worldwide. VZV is the causative agent of chickenpox (varicella) during primary infection and can later reactivate to cause shingles (herpes zoster [HZ]) [7,66]. Approximately 90% of the global population is estimated to become infected with VZV after reaching adulthood. Following primary infection, VZV establishes a latency period in the sensory ganglia, particularly the dorsal root and cranial nerve ganglia [67]. During latency, the virus remains dormant within neuronal cells and can persist for decades [67]. The reactivation of VZV can occur, and is often triggered by factors such as aging, immunosuppression, and stress, leading to HZ. The reactivation process involves the virus traveling along sensory nerves to the skin, causing the characteristic painful rash of shingles. Immunosuppressive therapies, including corticosteroids and biologics, can increase the risk of VZV reactivation by impairing the host immune response and allowing the dormant virus to become active again [67]. 

### 5.2. Biologics and Reactivation

Anti-TNF therapy can lead to the reactivation of VZV, but the associated risk is contentious. In a retrospective cohort study, several European studies indicated that anti-TNF treatment was significantly associated with an increased risk of HZ compared to non-biological disease-modifying antirheumatic drugs (DMARDs) [68,69,70]. In contrast, studies from the United States have not found an increased risk of HZ in patients receiving anti-TNF therapy compared to those receiving non-biologic DMARDs [71,72]. These conflicting results may be due to the differences in corticosteroid therapy practices between physicians in Europe and the United States [68,73].

Anti-CD20 monoclonal antibodies increase the risk of VZV reactivation. A large phase III randomized clinical trial involving previously untreated patients with follicular lymphoma investigated the anti-CD20 antibodies rituximab and obinutuzumab [74]. The study reported severe VZV infections in 8 of 597 patients (1.3%) in the rituximab group and in 6 of 595 patients (1%) in the obinutuzumab group. In another global phase III study, the Japanese subgroup reported VZV infections in 2 of 58 patients (3.4%) in the rituximab group and in 9 of 65 patients (13.8%) in the obinutuzumab group [75].

JAK inhibitors are associated with a significantly high risk of infectious complications, particularly HZ. In a phase III randomized controlled trial comparing ruxolitinib with the standard treatment for polycythemia vera, HZ occurred in 6% of patients treated with ruxolitinib, whereas no cases were reported in the control group [76]. Furthermore, a phase IV post-marketing study involving 1144 patients revealed that HZ was the most common infectious complication, with an incidence rate of 8% [77]. Additionally, a meta-analysis conducted in 2017 investigating ruxolitinib-related infectious complications highlighted a substantially increased risk of HZ, with an odds ratio of 7.39 compared to controls [78].

Other therapies, such as abatacept and the CD38 agent (daratumumab) are also associated with HVZ reactivation. However, neither the crude incidence rate nor the adjusted hazard ratio differed significantly between the biological agents. [66,79].

In conclusion, biological therapies, particularly anti-CD20 monoclonal antibodies and JAK inhibitors, are associated with a risk of VZV reactivation. Anti-TNF therapies exhibit mixed results with respect to an increased risk of HZ. Other biologics such as abatacept and anti-CD38 drugs also pose a risk for reactivation. Comprehensive screening and preventive measures are essential to effectively manage these risks.

## 6. Cytomegalovirus (CMV)

### 6.1. Pathogenesis and Latency

CMV, a member of the herpesvirus family, is highly prevalent worldwide. CMV infection can be asymptomatic in healthy individuals, but may cause severe disease in immunocompromised patients and neonates. Reports estimate that 66–90% of adults have been infected with CMV worldwide, with the prevalence varying by region and socioeconomic status [80,81]. Following primary infection, CMV establishes latency in various cells including monocytes, macrophages, and endothelial cells. During latency, the virus remains dormant within these cells and persists throughout the lifetime of the host. The reactivation of CMV can occur especially in the context of immunosuppression, such as in organ transplant recipients, individuals with human immunodeficiency virus (HIV)/acquired immunodeficiency syndrome (AIDS), and patients undergoing chemotherapy. Reactivation involves the virus replicating and shedding, which can lead to symptomatic disease, including CMV retinitis, pneumonitis, colitis, and hepatitis [82,83].

### 6.2. Biologics and Reactivation

Alemtuzumab, a CD52-receptor-targeting antibody that depletes T and B cells, has been approved for the treatment of active relapsing–remitting multiple sclerosis and steroid-refractory severe acute graft-versus-host disease (aGVHD) [84,85]. Severe immunosuppression, which can lead to CMV reactivation, is the most potent side effect of alemtuzumab. A single-center study evaluated 54 patients with various hematological malignancies who underwent allogeneic hematopoietic cell transplantation and were treated with alemtuzumab for steroid-refractory grade III or IV aGVHD. Among the patients, 35 (65%) exhibited positive CMV status in the donor and/or recipient. This study included three cases of CMV-mediated colitis and one case of CMV pneumonia [85].

Patients treated with the anti-CCR4 antibody, mogamulizumab, exhibit increased risk of CMV reactivation. In the FAERS database, 17 cases of CMV-related infections have been reported in patients treated with mogamulizumab compared to the 12,849 cases reported in patients treated with other medications. The calculated ROR was 55.89 (*p* < 0.001; 95% CI: 34.31–91.06) [57].

Anti-CD20 monoclonal antibodies such as rituximab also increase the risk of CMV reactivation; however, the relative risk is lower than that of mogamulizumab and alemtuzumab [57]. Prospective studies on the effect of anti-TNF therapy on viral reactivation in patients with RA or Crohn’s have not found any evidence of systemic CMV reactivation [86,87]. Clinical reports have also indicated only a few cases of severe CMV reactivation in patients undergoing anti-TNF therapy, including instances of CMV retinitis, CMV hepatitis, and disseminated infection [88].

## 7. Other Herpesviruses

Epstein–Barr virus (EBV) primarily targets the B lymphocytes, integrating into their genome and immortalizing the cells. During the latent phase of EBV infection, the immune system prevents viral reactivation through effective cellular immune responses [89]. Healthy individuals typically do not exhibit symptoms of EBV reactivation; however, immunocompromised individuals exhibit symptoms similar to those of initial EBV infection [89]. Although most EBV-infected individuals do not exhibit any symptoms, this virus can contribute to the onset of lymphoid and epithelial malignancies. Posttransplant lymphoproliferative disorder (PTLD) is a lymphoid or plasmacytic proliferative disease associated with immunosuppressive therapy following organ transplantation. EBV reactivation under immunosuppression is a significant risk factor for PTLD [90]. Belatacept (Nulojix) is a selective T-cell co-stimulation blocker that inhibits CD28-mediated T-cell co-stimulation by binding to CD80/86 ligands. It has been approved by the FDA for use in combination therapy to prevent kidney transplant rejection in patients seropositive for EBV. However, in a clinical trial, 0–4% of patients treated with belatacept developed PTLD [91].

Herpes simplex virus (HSV) and VZV belong to the same subgroup of herpesviruses (*Alphaherpesvirinae*) [67]. However, the risk of HSV reactivation is lower with biologics compared to VZV. Severe HSV diseases, including HSV encephalitis, HSV esophagitis, and disseminated cutaneous infections, have been reported in patients receiving TNFα-targeted therapies [92,93,94,95]. In a phase III randomized controlled trial investigating the effects of the anti-CD20 antibody, ocrelizumab, on primary progressive multiple sclerosis, oral HSV reactivation was more common in patients treated with ocrelizumab than in those receiving the placebo [96].

## 8. Polyomaviridae Family

### 8.1. Pathogenesis and Latency

*Polyomaviridae* is a family of small non-enveloped DNA viruses that infect various vertebrate hosts, including humans. Polyomaviruses are typically associated with asymptomatic infections; however, they can cause serious diseases under specific conditions. Human polyomaviruses, such as the BK virus (BKV) and JC virus (JCV), are widespread and usually acquired during childhood. Once acquired, these viruses can cause persistent infections in the host [97,98,99].

Polyomaviruses can remain latent in the kidneys, lymphoid tissues, and other organs throughout the host life. Reactivation of these viruses can occur in immunocompromised individuals, leading to various clinical manifestations. For instance, BKV reactivation is commonly associated with nephropathy in renal transplant patients, whereas JCV reactivation leads to progressive multifocal leukoencephalopathy (PML), a potentially fatal demyelinating disease of the central nervous system [98,99]. Immunosuppressive therapies, including those used for organ transplantation and autoimmune diseases, significantly increase the risk of polyomavirus reactivation.

### 8.2. Biologics and Reactivation

Treatment with biological agents is associated with the reactivation of JCV. Efalizumab, also known as raptiva, is a monoclonal antibody targeting CD11a that is used to treat psoriasis. However, as three of four patients receiving raptiva developed PML and died in previous studies, the drug has been withdrawn from the market [100,101]. Natalizumab (Tysabri) is a humanized monoclonal antibody targeting the alpha-4 integrin chain that is used to treat multiple sclerosis and Crohn’s disease [101,102]. Approximately 400 documented cases of PML associated with natalizumab use were reported from 2005 to August 2013 [100,101]. Alemtuzumab targets the CD52 antigen, resulting in the destruction of B and T cells. It has been approved by the FDA for the treatment of B-cell chronic lymphocytic leukemia. Recently, 14 cases of PML have been reported in patients treated with alemtuzumab [103]. Some patients develop PML after receiving rituximab for the treatment of RA, non-Hodgkin lymphoma, and chronic lymphocytic leukemia [104,105]. Notably, alemtuzumab does not increase the risk of BKV infection, but it reduces immunosuppression; hence, switching to calcineurin inhibitor-free regimens is the most viable therapeutic option [8,106].

## 9. Retroviruses

Retroviruses are a unique class of RNA viruses characterized by their distinct replication mechanisms [107]. Their genomes consist of single-stranded RNA; however, after infecting the host, these viruses use reverse transcriptase to reverse-transcribe their RNA into DNA, which is subsequently integrated into the host genome. Among the retroviruses, HIV and human T-cell leukemia virus type 1 (HTLV-1) are mostly associated with human pathogenicity. Both primarily infect the CD4+ T lymphocytes [107,108,109,110]. 

Several weeks after the initial HIV infection, this virus enters the acute phase, followed by a latent period. Despite the absence of overt symptoms during this stage, HIV continues to replicate within the body and progressively weakens the immune system. When the number of CD4+ T cells decreases to a critical threshold, the patient’s immune system becomes severely compromised, making them more susceptible to opportunistic infections and even cancer. This stage is referred to as the AIDS stage [111].

The overall safety profile of biological agents is generally favorable for HIV-infected individuals. A systematic review of 112 studies up to the year 2022 examined the use of biological therapies in 179 patients with HIV. The findings indicated that almost all categories of biological agents exhibit favorable safety profiles with minimal adverse events of mostly mild severity [112]. Therefore, HIV screening is necessary before initiating biological therapy, especially for patients engaging in high-risk behaviors. For HIV-positive patients, biological therapy should be administered only to those patients in a stable condition with CD4 cell counts > 200/mL [88].

Globally, HTLV-1 infects approximately 5–10 million people, with the vast majority being asymptomatic carriers. The most common diseases associated with HTLV-1 infection are adult T-cell leukemia/lymphoma (ATL), HTLV-1-associated myelopathy, and HTLV-1 uveitis [113,114,115,116,117,118]. TNF-α inhibitors do not affect the reactivation of HTLV-1 [119,120,121,122]. Some patients develop ATL after receiving the rituximab, cyclophosphamide, doxorubicin hydrochloride (hydroxydaunorubicin), vincristine sulfate (oncovin), and prednisone combination chemotherapy for B-cell lymphoma-associated hemophagocytic syndrome [123]. One patient with RA carrying the HTLV-1 virus was previously diagnosed with ATL after long-term treatment with TCZ [114,124]. Another case report also documented a patient with RA who experienced an exacerbation of HTLV-1-associated myelopathy and HTLV-1 uveitis following treatment with TCZ [125]. However, no direct evidence linking the development of HTLV-1-associated diseases to the use of biological agents has been reported to date.

## 10. Conclusions

Biological therapies are increasingly used for autoimmune diseases, cancer treatment, and organ transplantation. These therapies target specific molecular and cellular pathways, significantly improving patient prognosis. However, different biological therapies exhibit varying risks of reactivating latent pathogens owing to their distinct targets. Therefore, associations between different biological therapies and viral infections must be explored to better understand and manage the risk of pathogen reactivation. In clinical practice, potential risks and benefits should be considered to select the appropriate biological therapy. Furthermore, preventive measures must be developed to decrease the risk of latent pathogen reactivation.

## Figures and Tables

**Table 1:** List of biological therapies, their immunosuppressive mechanisms, and clinical applications.

Category	Representative Drugs	Primary Target	Immunosuppressive Mechanism	Clinical Applications	
				Autoimmunity Diseases	Cancers
TNF-α Inhibitors	Infliximab (Remicade), Adalimumab (Humira), Etanercept (Enbrel)	TNF-α	Inhibits TNF-α, reducing inflammation	Rheumatoid arthritis, Crohn’s disease, Psoriasis	-
B-Cell Targeting Agents	Rituximab (Rituxan), Obinutuzumab (Gazyva), Ofatumumab (Arzerra)	CD20	Depletes B cells, impairing humoral immune response	Rheumatoid arthritis, Systemic lupus erythematosus, Vasculitis	Non-Hodgkin lymphoma, Chronic lymphocytic leukemia
Checkpoint Inhibitors	Pembrolizumab (Keytruda), Nivolumab (Opdivo), Ipilimumab (Yervoy)	PD-1, PD-L1, CTLA-4	Activates the immune system, potentially causing immune dysregulation	-	Melanoma, Lung cancer, Renal cell carcinoma, Hodgkin’s lymphoma, Head and neck squamous cell carcinoma
IL-6 Inhibitors	Tocilizumab (Actemra), Sarilumab (Kevzara)	IL-6	Inhibits IL-6, affecting acute phase response and systemic immune reactions	Rheumatoid arthritis, Giant cell arteritis, Systemic juvenile idiopathic arthritis	-
IL-17 Inhibitors	Secukinumab (Cosentyx), Ixekizumab (Taltz)	IL-17	Inhibits IL-17, affecting neutrophil recruitment and function	Psoriasis, Psoriatic arthritis, Ankylosing spondylitis	-
JAK Inhibitors	Tofacitinib (Xeljanz), Baricitinib (Olumiant), Upadacitinib (Rinvoq)	JAK-STAT signaling pathway	Inhibits multiple cytokines signaling pathways, broadly suppressing the immune system	Rheumatoid arthritis, Psoriatic arthritis, Ulcerative colitis	-
CCR4 Inhibitors	Mogamulizumab (Poteligeo)	CCR4	Depletes Tregs and Th2 cells, impairing immune regulatory functions	-	Adult T-cell leukemia/lymphoma, Cutaneous T-cell lymphoma
IL-23 Inhibitors	Guselkumab (Tremfya), Risankizumab (Skyrizi), Tildrakizumab (Ilumya)	IL-23	Inhibits IL-23, affecting Th17 cell functions	Psoriasis, Crohn’s disease	-
CD38 Inhibitors	Daratumumab (Darzalex), Isatuximab (Sarclisa)	CD38	Broadly depletes CD38-positive cells, impairing immune surveillance	-	Multiple myeloma
T-Cell Costimulation Blockers	Abatacept (Orencia) Belatacept (Nulojix)	CD80/CD86	Inhibits T-cell costimulatory signals, weakening T-cell-mediated immune responses	Rheumatoid arthritis, Organ transplantation, Psoriasis, Psoriatic arthritis	-
IL-1 Inhibitors	Anakinra (Kineret), Canakinumab (Ilaris)	IL-1	Inhibits IL-1, reducing inflammation	Rheumatoid arthritis, Neutrophilic dermatoses	Multiple myeloma
IL-12/IL-23 Inhibitors	Ustekinumab (Stelara)	IL-12/IL-23 p40 subunit	Inhibits IL-12 and IL-23, reducing Th1 and Th17 responses	Psoriasis, Crohn’s disease	-
IL-4/IL-13 Inhibitors	Dupilumab (Dupixent)	IL-4Rα	Inhibits IL-4 and IL-13 signaling, reducing inflammation	Atopic dermatitis, Eosinophilic esophagitis, Asthma	-
IL-5 Inhibitors	Mepolizumab (Nucala), Reslizumab (Cinqair), Benralizumab (Fasenra)	IL-5	Inhibits IL-5, reducing eosinophil activity	Eosinophilic asthma	-
Complement Inhibitors	Eculizumab (Soliris)	C5	Inhibits complement component C5, preventing complement-mediated damage	Paroxysmal nocturnal hemoglobinuria, Atypical hemolytic uremic syndrome	-
Integrin Inhibitors	Natalizumab (Tysabri), Vedolizumab (Entyvio)	α4-integrin, α4β7-integrin	Inhibits integrin-mediated cell adhesion, reducing immune cell infiltration	Multiple sclerosis, Crohn’s disease, Ulcerative colitis	-
CD52 Inhibitors	Alemtuzumab (Lemtrada)	CD52	Depletes CD52-positive cells, broadly suppressing the immune system	Multiple sclerosis	Chronic lymphocytic leukemia
GM-CSF Inhibitors	Sargramostim (Leukine)	GM-CSF	Modulates GM-CSF signaling, affecting immune cell activation	Bone marrow transplantation	Chronic myelogenous leukemia
IL-2 Receptor Antagonists	Basiliximab (Simulect), Daclizumab (Zinbryta)	IL-2Rα (CD25)	Blocks IL-2 signaling, reducing T-cell proliferation	Prevention of organ transplant rejection, Multiple sclerosis	-
RANKL Inhibitors	Denosumab (Prolia, Xgeva)	RANKL	Inhibits RANKL, reducing osteoclast activity	Osteoporosis	Bone metastases
Anti-VEGF Antibodies	Bevacizumab (Avastin), Ranibizumab (Lucentis)	VEGF	Inhibits VEGF, reducing angiogenesis	Age-related macular degeneration	Metastatic colorectal cancer, Non-small cell lung cancer, Glioblastoma, Ovarian cancer, Renal cell carcinoma

Abbreviations: TNF-α: Tumor Necrosis Factor-alpha; CD20: Cluster of Differentiation 20; PD-1: Programmed Death-1; PD-L1: Programmed Death-Ligand 1; CTLA-4: Cytotoxic T-Lymphocyte-associated protein 4; IL-6: Interleukin-6; IL-17: Interleukin-17; JAK-STAT signaling pathway: Janus Kinase-Signal Transducer and Activator of Transcription signaling pathway; CCR4: C-C Motif Chemokine Receptor 4; IL-23: Interleukin-23; CD38: Cluster of Differentiation 38; CD80/CD86: Cluster of Differentiation 80/86; IL-1: Interleukin-1; IL-12/IL-23 p40 subunit: Interleukin-12/Interleukin-23 p40 subunit; IL-4Rα: Interleukin-4 Receptor alpha; IL-5: Interleukin-5; C5: Complement Component 5; α4-integrin, α4β7-integrin: alpha-4 Integrin, alpha-4 beta-7 Integrin; CD52: Cluster of Differentiation 52; GM-CSF: Granulocyte-Macrophage Colony-Stimulating Factor; IL-2Rα (CD25): Interleukin-2 Receptor alpha (Cluster of Differentiation 25); RANKL: Receptor Activator of Nuclear Factor kappa-B Ligand; VEGF: Vascular Endothelial Growth Factor.

**Table 2:** Risk of HBV reactivation associated with planned biologic therapy based on baseline HBV status.

Category	HBsAg+ Risk	HBsAg-/Anti-HBc+ Risk
Anti-CD20	High	Moderate
Anti-TNF	High	Low
Anti-IL-6	High	Moderate
Other cytokine inhibitors	High	Moderate
Anti-CCR4	Moderate	Moderate
Immune checkpoint inhibitors	High	Low
JAK inhibitors	High	High

Definitions of risk level: high risk: anticipated incidence of HBVr > 10%; moderate risk: anticipated incidence of HBVr 1–10%; low risk: anticipated incidence of HBVr < 1%.

## Data Availability

All data related to this study are presented and published here.
